# Is an Absolute Barrier Imposed?

**Published:** 1920-03-20

**Authors:** 


### SCRAPPING THE NURSE.—II.

### Is an Absolute Barrier Imposed?

THE point that I desire to emphasise, with regard to the training of health visitors, is that the privilege granted to trained nurses of being obliged to train at a Polytechnic Institute for one instead of for two years is of so little value that it is negligible.

I do not know how many people agree with me that trained nurses should take a very large and a very active part in the reconstruction of the national health. Therein lies the gravamen of the whole question. I hold that a nurse's hospital training not only fits her, but entitles her, to major participation, and I contend that the Ministry's requirements, which I regard as unreasonable, form an insuperable barrier.

A trained nurse is, as a general rule, at least four- or five-and-twenty years of age. She has served a hard apprenticeship in learning her profession. She is, and must be, a wage-earner. In many cases she has not only to support herself, but to contribute to the support of others. Is it, in the circumstances, reasonable to suggest that she should resign her appointment and start fresh as a speculative student? Even if she had the means, it is extremely doubtful that she would have the heart, or, if you please, the enterprise, to contemplate a new career.

Wittingly or unwittingly, accidentally or by design, the regulations of the Ministry have the obvious effect of putting her outside the pale.

### EDUCATIONAL INSTITUTIONS.

I want to know what is wrong with a hospital that it cannot be equipped and staffed so as effectually to impart the necessary training? Dr. Addison's letter to me offers no explanation. It is simply a dogmatic statement that it cannot be done, and the regulations confer a monopoly on institutions connected with Universities.

I venture to suggest that my question demands an answer. Here is a summary of the regulations setting out the curriculum:

A student will not be eligible for admission to the course unless she satisfies the authorities of the institution that—

i. She is over eighteen years of age at the date of the commencement of the course.

ii. Her previous education has been such that she is likely to profit by the course.

The course must provide for both theoretical instruction and practical training in about equal proportions.

The theoretical instruction should include instruction in—

i. Elementary physiology, and the structure of the body. ii. Methods of artisan cookery and household management. iii. Hygiene. iv. Infectious and communicable diseases. v. Maternity, infant and child welfare. vi. Elementary economics and social problems.

The practical training must be designed to illustrate the theoretical instruction and to give the students a first-hand personal knowledge of the various aspects of their future work.

Every one of these subjects is taught in well-equipped hospitals, and taught in a way that no University institution can approach. The nurse must be able to cook not merely what would suit an artisan, but the sick poor. And, as for social problems, hospital administration is nothing else than one huge social problem which committee, medical and nursing staffs are trying all their time to solve.

Will anybody seriously compare the worth of a Polytechnic student after two years of training with that of a trained nurse after three, or, as it used to be in practice, four, as an officer engaged in national health work? And yet the nurse is to be the exception and the schoolwoman the rule.

### THE HOSPITAL ATTITUDE.

I have not discussed, or even considered, the hospital attitude. Possibly there is none, as the hospital is declared out of bounds. But I should say that if it were put on a par with the Polytechnic it would show a singularly unbusinesslike talent if it did not immediately enter into the arena. Hospitals like new blood in their nursing staffs. They do not desire that sisters and nurses should remain at their posts until they retire because of old age. The health-visiting field of employment would ensure this. Again, the intelligent Committee, and certainly, without exception, every Matron, likes a good type of candidate probationer, socially and educationally. With bright prospects, the young women who are now deserting nursing for more ambitious employment would clamour for admission.

### THE SUPREME TEST.

The supreme test is fitness. There are Cabinet Ministers who have never been educated at a University or at a University Institution. It is surely possible for the Board of Education and the Ministry of Health to find a means of ascertaining whether a candidate health visitor is properly educated and trained for her work without the aid of a certificate that she has attended so many lectures at a specified institution. What is wrong with an oral and written examination? And has the College of Nursing, as an educational solvent of the nurse's difficulties, nothing to say in the matter?

IERNE.

